# Development of KASP Molecular Markers and Candidate Gene Mining for Heat Tolerance-Related Traits in *Gossypium hirsutum*

**DOI:** 10.3390/genes16101154

**Published:** 2025-09-28

**Authors:** Zhaolong Gong, Ni Yang, Shiwei Geng, Juyun Zheng, Zhi Liu, Fenglei Sun, Shengmei Li, Xueyuan Li, Yajun Liang, Junduo Wang

**Affiliations:** 1 Xinjiang Cotton Technology Innovation Center, Xinjiang Key Laboratory of Cotton Genetic Improvement and Intelligent Production, National Cotton Engineering Technology Research Center, Cotton Research Institute of Xinjiang Uyghur Autonomous Region Academy of Agricultural Sciences, Urumqi 830091, China; 2 College of Bioscience and Biotechnology, Hunan Agricultural University, Changsha 410128, China; zhiliu@hunau.edu.cn

**Keywords:** *G. hirsutum*, high temperature, KASP, candidate gene

## Abstract

**Background**: High-temperature stress is one of the major abiotic stresses limiting cotton production. Identifying genetic loci and genes for heat tolerance is crucial for breeding heat-tolerant varieties. **Methods**: Given the complexity of heat tolerance phenotypes in cotton, this study, which focused on resource materials, identified an A/C SNP mutation at position 5486185 on chromosome D06 within the heat tolerance interval through genome-wide association studies (GWAS) of natural *Gossypium hirsutum* populations. **Results**: A total of 308 resource materials were identified and evaluated for their heat tolerance phenotypes over two years of field research. Kompetitive allele-specific PCR (KASP) molecular markers were developed on the basis of the D06-5486185 SNP to characterize the heat tolerance phenotypes of these 308 resource materials. Genotyping for heat tolerance-related traits and agronomic traits was also performed. Materials with the C/C haplotype at position D06-5486185 presented increased heat tolerance (higher pollen viability (PV), leaf area (LA), chlorophyll (Chl) and number of bolls on the third fruit branch (FB3) and a lower number of dry buds (DBs) and drop rate (DR)) without negatively impacting key yield traits. This locus is located in the intergenic region of two adjacent bZIP transcription factor genes (*GH_D06G0408* and *GH_D06G0409*). Expression analysis revealed that the expression levels of these two genes were significantly greater in heat-tolerant accessions (C/C type) than in sensitive accessions and that their expression levels were significantly correlated with multiple heat-tolerant phenotypes. **Conclusions**: In summary, this study developed a Kompetitive Allele Specific PCR (KASP) marker associated with heat tolerance in *G. hirsutum* and identified two key heat tolerance candidate genes. These results provide an efficient marker selection tool and important genetic resources for the molecular breeding of heat-tolerant *G. hirsutum*, laying an important foundation for further establishing a molecular marker-assisted breeding system for heat tolerance in *G. hirsutum*.

## 1. Introduction

In the context of global climate change, frequent high-temperature stress has become a major abiotic stress factor affecting crop production [1]. Studies have shown that for every 1 °C increase in the global average temperature, the yields of major crops—wheat, rice, corn, and soybeans—will decrease by 6%, 3.2%, 7.4%, and 3.1%, respectively [2]. Temperatures exceeding 35 °C can cause cotton anthers to dehisce prematurely and reduce pollen viability, resulting in impaired pollination and reduced boll set [3]. Moreover, high temperatures intensify respiratory consumption, creating an imbalance between the supply and demand of photosynthates. This metabolic disruption leads to the abscission of buds, flowers, and bolls, ultimately resulting in yield loss. More seriously, global warming is projected to cause a reduction in total crop yields of approximately one-third by 2050 [4]. Cotton, a major cash crop and a major source of natural textile fiber, also faces severe challenges from high-temperature stress [5]. Xinjiang, China, with its unique advantages in terms of light and heat resources, has become China’s largest high-quality cotton production base [6]. However, persistent high temperatures in the region from July to August, which coincided with the cotton flowering and boll-bearing period, severely hampered cotton production [7]. High temperatures significantly reduced pollen vitality, caused young buds to dry out and fall off, and particularly sharply reduced the number of bolls formed on the upper third fruiting branches, resulting in a large number of bud and boll dropouts [8]. High temperatures also reduce cotton photosynthesis efficiency, water use efficiency, and nutrient absorption efficiency, thereby inhibiting normal plant growth and development. Furthermore, high temperatures can cause reproductive disorders such as pollen abortion and anther failure, premature flowering, and a shortened growing season, ultimately leading to reduced fiber quality and yield [9]. These heat stress effects have become a major bottleneck restricting high-yield and high-quality cotton. Therefore, breeding new heat-resistant cotton varieties is key to addressing the challenges of climate change and overcoming the stagnant yield situation.

Traditional cotton breeding relies primarily on field phenotyping of large populations, which not only is time-consuming and labor-intensive but also makes accurate selection of superior lines difficult [10]. With the significant reduction in sequencing costs and the rapid development of molecular biology techniques, marker-assisted selection (MAS) has become a mainstream technology in modern breeding because of its advantages, such as short turnaround time and wide applicability, significantly improving breeding efficiency [11]. Currently, MAS has been widely applied in major crops such as rice, wheat, corn, and rapeseed, but its application is still focused primarily on improving yield and quality traits, with relatively little research and application on traits such as stress tolerance and environmental adaptability [12]. Owing to their widespread distribution across the genome, single-nucleotide polymorphisms (SNPs), which are third-generation molecular markers, have provided a powerful boost in the development of molecular breeding [13]. Among them, Kompetitive Allele Specific PCR (KASP) combines the advantages of PCR amplification and fluorescence detection to enable high-throughput genotyping of large populations, providing a flexible and cost-effective solution for the large-scale conversion and application of SNP markers [14]. Compared with Sanger sequencing and high-throughput sequencing, KASP is more efficient at identifying polymorphic loci; this technology has achieved significant success in a variety of crops [14,15,16]. For example, KASP markers were developed based on a QTL associated with stripe rust resistance in wheat, thereby facilitating the introduction of this resistance QTL into new wheat varieties [17]. In rice breeding, researchers have used KASP technology to develop functional markers for three major genes affecting rice quality (*Wx*, *BADH2* and *ALK*), successfully breeding superior restorer lines with improved rice quality [18]. In wheat disease resistance breeding, three KASP markers were developed through QTL mapping in a recombinant inbred line population constructed from parents resistant to wheat streak mosaic virus, effectively distinguishing between different resistant and susceptible genotypes [19]. Furthermore, in mango breeding, five molecular markers associated with important traits were validated via genome-wide association studies (GWASs) and KASP technology [20]. These successful cases fully demonstrate the broad application prospects of KASP technology in crop molecular breeding.

Currently, the selection of heat-tolerant *G. hirsutum* varieties still relies primarily on phenotypic identification and lacks efficient and reliable molecular markers [3]. Research has shown that combining MAS technology with conventional breeding methods can significantly improve the accuracy and efficiency of breeding selection and shorten the breeding cycle [21]. Therefore, there is an urgent need to identify molecular markers closely associated with key heat tolerance traits to support the rapid identification and targeted breeding of superior heat-tolerant cotton varieties. To this end, this study systematically evaluated the tolerance phenotypes of 308 *G. hirsutum* germplasm accessions under two consecutive years of natural high-temperature stress in the field. On the basis of previously mapped heat tolerance quantitative trait loci (QTLs), a stable and efficient molecular marker (KASP) for heat tolerance traits was successfully developed (D06-5486185 SNP). Further expression analysis and association analysis identified two candidate genes associated with heat-tolerant *G. hirsutum*. These results lay an important foundation for establishing a molecular marker-assisted breeding system for heat-tolerant *G. hirsutum.*

## 2. Materials and Methods

### 2.1. Plant Materials

A total of 308 *G. hirsutum* accessions were used for molecular marker development in this study, all of which were collected and provided by the Institute of Economic Crops, Xinjiang Academy of Agricultural Sciences. Phenotypic characterization of heat tolerance and agronomic traits of these 308 accessions was conducted in 2022 and 2023 at the Xinjiang Academy of Agricultural Sciences Cotton Comprehensive Experimental Station, 16th Regiment, First Division, Aral City, Xinjiang (40°29′ N, 80°50′ E). The planting density was 15,500 plants/mu in a six-row (10 + 66 + 10 + 66 + 10) cm planting pattern with drip irrigation under mulch, three replicates in randomized blocks, and a row length of 4.5 m. Base fertilizer was applied in a single dose before sowing, consisting of 600 kg/ha of diammonium phosphate, 180 kg/ha of urea, and 120 kg/ha of potassium fertilizer. Manual dibbling was used for sowing. During the growth period, intertillage and weeding were performed 7 times, followed by 10 drip irrigations (approximately 3300 m^3^/ha), 10 fertilizations coupled with irrigation, and 8 chemical growth regulations. Thirteen important traits, including plant height (PH), height of the first fruiting node (HFNFH), number of effective fruiting branches (EFB), number of bolls (NB), single boll weight (SBW), lint percentage (LP), pollen viability (PV), leaf area (LA), chlorophyll (Chl), number of dry buds (DBs), abscission rate of the last three fruit branch (FB3), number of bolls on the last three fruit branch (CB3) and drop rate (DR), were investigated.

Following two years of field-based high-temperature tolerance screening, we selected ten heat-tolerant and ten heat-sensitive accessions to analyze the expression patterns of candidate genes. All uniform seeds were sterilized in a 15% hydrogen peroxide solution for 4 h and then rinsed twice with sterile water. The seeds were transferred to germination boxes (12 cm long × 12 cm wide × 6 cm high) filled with sterilized sand. Ten seeds were sown evenly in each box at a depth of 2 cm. After sowing, 300 mL of deionized water was added to the box to saturate the sand. Two hundred milliliters of water were added every two days, and the germination boxes were placed in an illuminated incubator. The growth conditions included a photoperiod (16 h daytime, 8 h nighttime), temperature (28 °C daytime, 20 °C nighttime), optical density (300 µmol m^−2^ s^−1^), and relative humidity (75%). When the cotton seedlings reached the three-leaf stage, they were subjected to 40 °C heat stress in a lighted incubator for 24 h and then sampled [22]. Three replicates were used for each treatment.

### 2.2. Development of KASP Markers

DNA was extracted from the leaves of 308 *G. hirsutum* accessions via a modified CTAB method. This experiment commissioned the LGC high-throughput genotyping platform of Xinjiang Aidesen Biotechnology Co., Ltd. (Urumqi, China) to perform KASP detection at the D06-5486185 SNP locus (primers are provided in Table S1). The KASP assay steps were as follows: DNA samples were transferred to a 96-well plate via a Replikator instrument. The plate containing DNA was then dried in a PRIME Incubator at 65 °C for 30 min. PCR was performed on a dried 96-well plate using a Meridian instrument. After the reaction mixture was added, the plate was sealed and centrifuged at 4000 rpm for 5 min before PCR was performed. After the plate cooled, the plate was read via a Pherastar microplate reader. Typing clusters were visualized and exported via SNPviewer software (version 0.9.2) [23]. In this experiment, the initial attempt at KASP genotyping achieved a success rate of 29.87%, with 92 samples producing clear and interpretable clustering patterns following the first amplification round. If clear typing results were not obtained, additional PCRs were performed for further amplification.

### 2.3. qRT—PCR

Total RNA was extracted via the RNAprep Pure Polysaccharide and Polyphenol Plant Total RNA Extraction Kit (Tiangen, Beijing, China). The concentration of each RNA sample was determined via a NanoDrop 2000 spectrophotometer (Thermo Fisher Scientific, Waltham, MA, USA). The RNA was reverse transcribed to generate cDNA via the M-MLV RTase cDNA Synthesis Kit (TaKaRa, Kyoto, Japan). qRT—PCR analysis was performed using a Roche LC480 instrument (Roche Diagnostics GmbH, Mannheim, Germany) and SYBR Green (TaKaRa, Kyoto, Japan). The reaction volume was 20 μL. The reaction procedure consisted of 95 °C initial denaturation for 30 s, followed by 35 cycles of denaturation at 95 °C for 5 s, annealing at 60 °C for 5 s, and extension at 72 °C for 30 s. Relative quantification was performed via the 2^−ΔΔCt^ method with the internal reference gene *Gh_UBQ7* [24]. Three biological replicates were used for each reaction. All primer information can be found in Table S1.

### 2.4. Data Analysis

Statistical analysis of the 13 traits was performed via Excel 2010 and the R programming language (Version 4.2.1), specifically including the describe function from the psych package, the corrplot package, and the ggplot2 package. One-way analysis of variance was used to determine the significance of differences between genotypes. Prior to conducting the ANOVA, the normality and homogeneity of variances of the data were assessed. Normality was evaluated using the Shapiro–Wilk test, while Levene’s test was employed to verify the homogeneity of variances across groups.

## 3. Results

### 3.1. Descriptive Statistics and Correlation Analysis of Population Phenotypes

**Figure 1:**
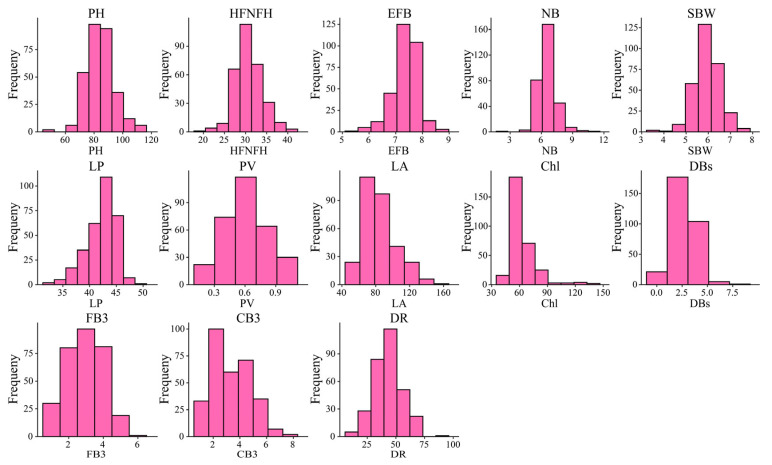
Frequency distribution histogram of all phenotypic traits.

Descriptive statistical analysis of the traits of 308 natural *G. hirsutum* accessions collected over a two-year survey was performed (Table 1). The coefficients of variation for each trait ranged from 6.59% to 46.85%, with the coefficients of variation exceeding 10% for all traits except LP, EFB, and SBW. The coefficients of variation for traits related to heat tolerance (Chl, LA, DR, PV, FB3, CB3, and DBs) all exceeded 23.25%. DBs had the highest coefficient of variation (46.85%), with an average of 2.54, a minimum of 0.20, and a maximum of 8.40. The skewness and kurtosis results revealed that all the traits followed a normal distribution (Figure 1). Furthermore, the mean, minimum, and maximum values of these traits also revealed significant variation among the accessions. These findings indicate that traits related to heat tolerance vary significantly within populations, providing important evidence for further genotyping of natural populations via KASP markers.

**Figure 2:**
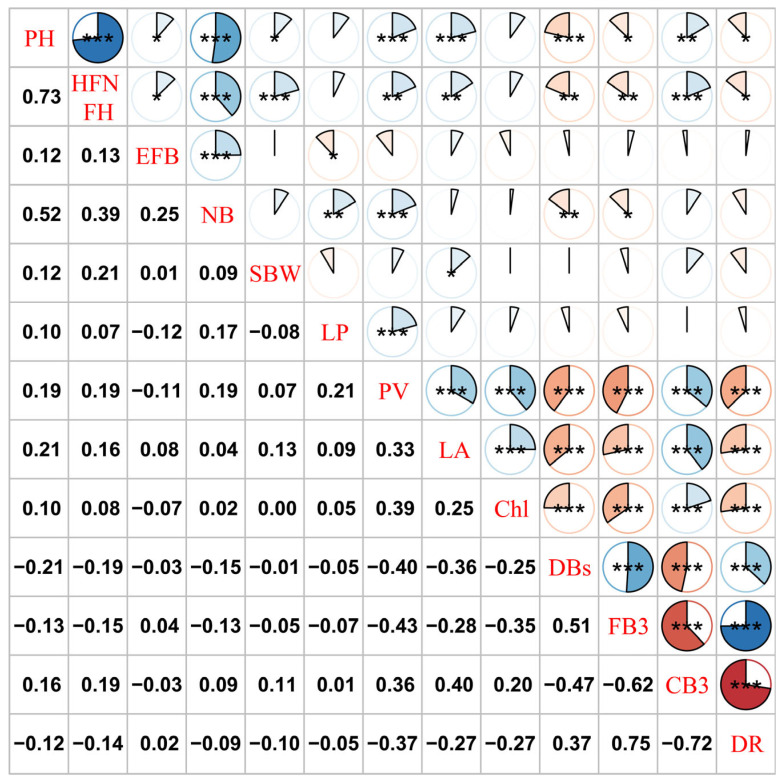
Correlation analysis of all phenotypic traits. Unmarked values indicate no significance. * *p* < 0.05, ** *p* < 0.01, *** *p* < 0.001.

To further explore the relationships among traits, correlation analysis was performed on various traits in 308 *G. hirsutum* accessions (Figure 2). PH, HFNFH, and NB were significantly positively correlated, with correlation coefficients greater than 0.5. HFNFH was significantly positively correlated with NB, with a correlation coefficient of 0.52. PV, LA, Chl, DBs, FB3, CB3, and DR were highly significantly correlated with each other. DB, FB3, and DR were significantly negatively correlated with PV, LA, Chl, and CB3, whereas DB, FB3, and DR were significantly positively correlated. The data for seven phenotypes (PV, LA, Chl, DBs, FB3, CB3, and DR) after high-temperature stress were clustered into one category, whereas the remaining traits were clustered into one category. These findings suggest that these seven phenotypes can reflect the response of cotton to heat stress from different perspectives.

### 3.2. Development of KASP Molecular Markers

**Figure 3:**
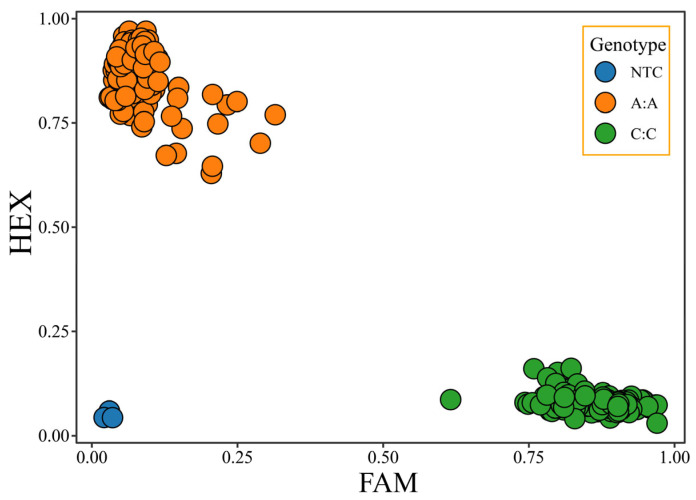
Scatter plot of the genotyping of 308 natural populations of G. *hirsutum* via KASP markers at SNP sites D06-5486185. Different colors represent different haplotypes.

Previous GWAS results on heat tolerance in *G. hirsutum* revealed that the A/C SNP mutation at position 5486185 on chromosome D06 can distinguish between susceptible and resistant accessions. To this end, two pairs of KASP primers were designed on the basis of the A/C mutation at position 5486185 on chromosome D06. The upstream primer F1 amplifies the A site at position D06-5486185, and the upstream primer F2 amplifies the C site at position D06-5486185. The downstream primer R serves as a universal primer. These two pairs of KASP primers were used to genotype 308 natural G. *hirsutum* accessions (Figure 3). Among the 308 natural *G. hirsutum* accessions, 170 accessions were of the A:A genotype, and 138 accessions were of the C:C genotype. The fluorescent cluster signals for each genotype clustered together in the genotyping results for the natural populations, indicating that the KASP marker at the D06-5486185 SNP is highly polymorphic and can distinguish between different haplotypes.

**Figure 4:**
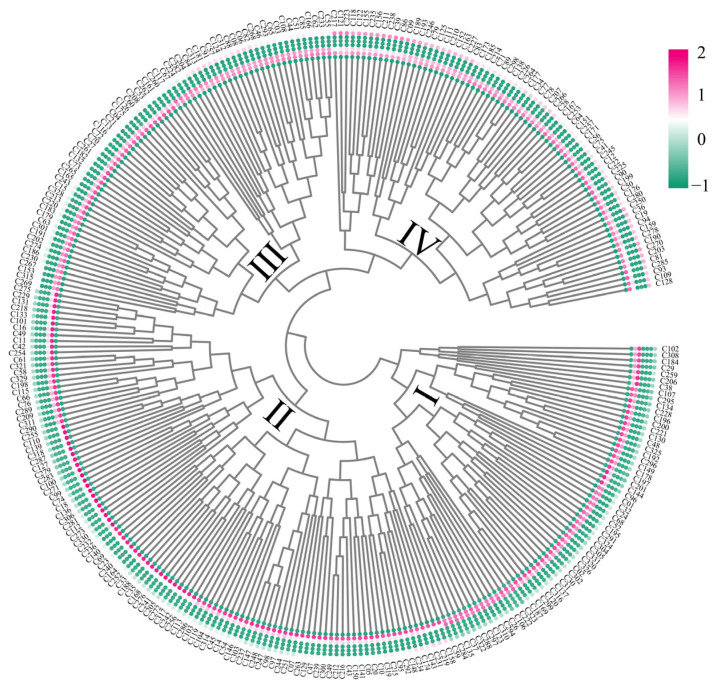
Cluster heatmap of the high-temperature resistance of 308 *G. hirsutum* resources.

On the basis of phenotypic indicators (including PV, LA, Chl, DBs, FB3, CB3, and DR) during the tetrad period after high-temperature stress, this study systematically evaluated the heat tolerance of 308 G. *hirsutum* accessions through cluster analysis (Figure 4). Cluster analysis divided the accessions into four categories: Category I, heat-tolerant accessions, totaling 62 accessions, of which 83.87% were C/C genotypes and 16.13% were A/A genotypes; Category II, relatively heat-tolerant accessions, totaling 106 accessions, of which 66.04% were C/C genotypes and 33.96% were A/A genotypes; Category III, heat-sensitive accessions, totaling 68 accessions, of which 14.71% were C/C genotypes and 85.29% were A/A genotypes; and Category IV, extremely heat-sensitive accessions, totaling 72 accessions, of which only 8.33% were C/C genotypes and 91.67% were A/A genotypes. Notably, among all 138 materials harboring the C/C genotype, the vast majority presented strong high-temperature tolerance, indicating that this haplotype is related to the high-temperature tolerance of *G. hirsutum.*

### 3.3. Genotype—Phenotype Association Analysis

**Figure 5:**
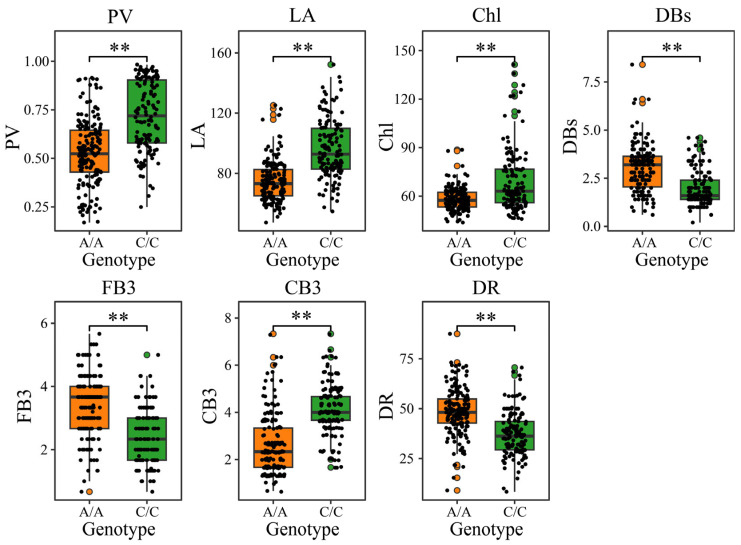
Difference analysis of different haplotypes of PV, LA, Chl, DBs, FB3, CB3 and DR after high-temperature stress, ** *p* < 0.01.

On the basis of SNP genotype data at the D06-5486185 locus across 308 G. *hirsutum* accessions, this study conducted association analyses with multiple heat tolerance-related phenotypes to systematically explore the association between this genotype and heat tolerance. The results revealed significant differences between the A/A and C/C haplotypes in PV, LA, Chl, DBs, FB3, CB3, and DR (Figure 5). Specifically, the C/C genotype presented greater PV, greater LA, higher Chl, and more FB3, whereas DBs, CB3, and DR were significantly lower than those of the A/A haplotype. These results consistently indicate that haplotype variation at the D06-5486185 locus is significantly associated with overall heat tolerance in *G*. *hirsutum* accessions and that the C/C genotype may serve as a potential selective marker for heat tolerance.

**Figure 6:**
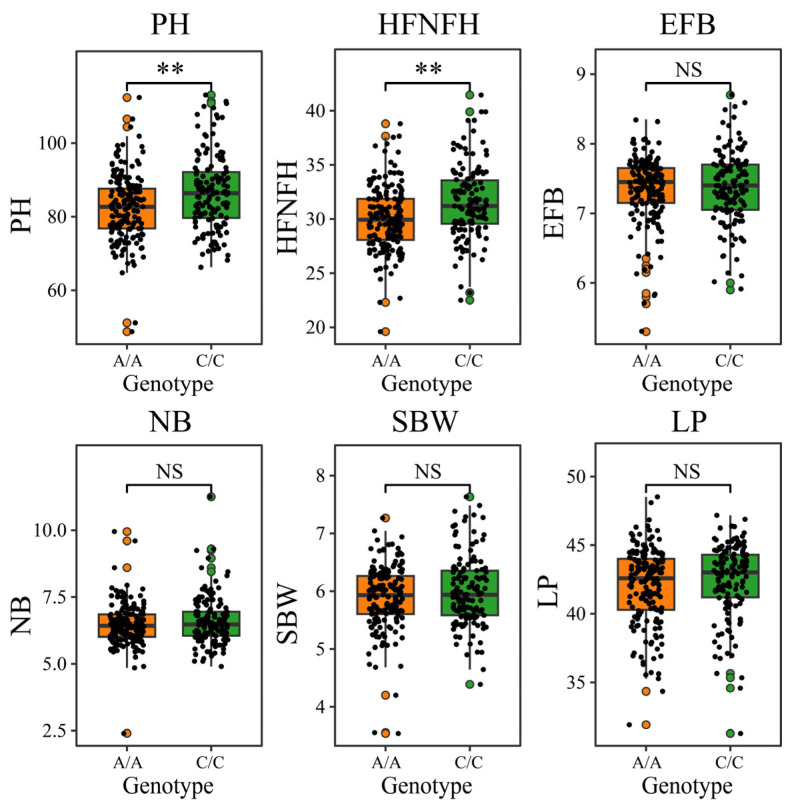
Difference analysis of different haplotypes for the PH, HFNFH, EFB, NB, SBW, and LP traits, ** *p* < 0.01.

Further analysis revealed that the relationships between different haplotypes at the D06-5486185 locus and agronomic and yield traits were not significant. The results revealed that, with the exception of pH and HFNFH, no significant differences were observed in other traits (including EFB, NB, SBW and LP). Compared with the other genotypes, the C/C genotype had a significantly greater pH and HFNFH (Figure 6). These results indicate that the genotype at the D06-5486185 SNP locus is significantly associated with multiple heat tolerance-related traits and that the C/C genotype maintains high heat tolerance without negatively impacting key yield traits such as EFB, NB, SBW and LP. Therefore, this locus can serve as a reliable molecular marker for the identification and screening of heat-tolerant *G. hirsutum* germplasm resources, providing an important theoretical basis and application foundation for molecular marker-assisted breeding of heat-tolerant cotton.

### 3.4. Screening of Candidate Genes

**Figure 7:**
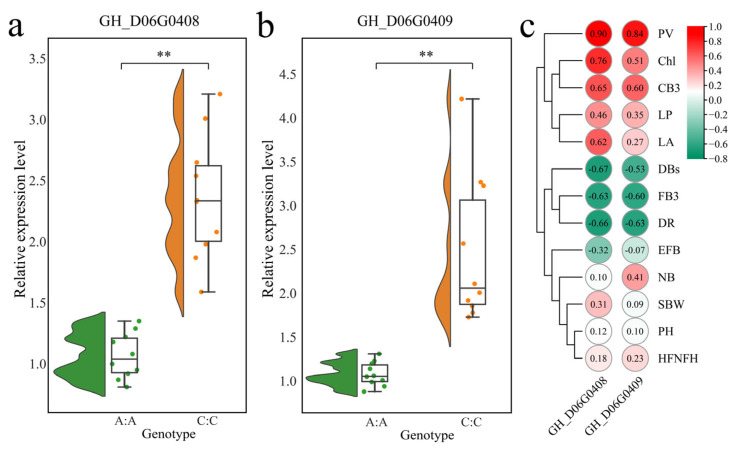
Analysis of candidate gene expression patterns and correlations with phenotypes. (**a**) Expression pattern analysis of *GH_D06G0408* in extreme accessions, (**b**) expression pattern analysis of *GH_D06G0409* in extreme accessions, (**c**) heatmap of correlations between *GH_D06G0408* and *GH_D06G0409* expression levels and phenotypes. ** *p* < 0.01.

To further identify candidate genes for heat tolerance in *G. hirsutum*, we analyzed locus 5486185 on chromosome D06 and found that it was located in the intergenic region between *GH_D06G0408* and *GH_D06G0409*. On the basis of these findings, we identified *GH_D06G0408* and *GH_D06G0409* as candidate genes for heat tolerance for further study. Annotation of homologous genes in *Arabidopsis thaliana* revealed that both genes encode bZIP transcription factors. We selected 10 heat-tolerant accessions (with the C/C haplotype at locus D06-5486185) and 10 heat-sensitive accessions (with the A/A haplotype at locus D06-5486185) to measure the expression levels of *GH_D06G0408* and *GH_D06G0409* (Figure 7a,b). The results revealed that the expression levels of both genes were significantly greater in the heat-tolerant accessions than in the heat-sensitive accessions. Further correlation analysis revealed that the expression levels of *GH_D06G0408* and *GH_D06G0409* were positively correlated with PV, Chl, and FB3 (correlation coefficients > 0.6) and negatively correlated with DBs, CB3, and DR (correlation coefficients < −0.5) (Figure 7c). In summary, we not only developed potential molecular markers for high-temperature tolerance breeding in *G. hirsutum* but also identified new candidate genes for high-temperature tolerance.

## 4. Discussion

High temperatures significantly impact crop traits such as growth period, morphology, yield, quality, and resistance [25]. Studies have shown that high temperatures significantly impact reproductive development, including the development of gametophytes, anthers, and pollen tubes, followed by physiological regulation [26]. In rice, high-temperature treatment (39 °C/30 °C) beginning at the microspore stage reduced spikelet fertility [27]. Morphological observations revealed that pollen viability in high-temperature-treated plants was lower than that in controls, with pollen exhibiting a normal round shape. When the temperature during millet flowering was between 26 °C and 36 °C, the number of seeds, plant weight, and yield decreased significantly [28]. This is primarily due to the high temperatures causing floret sterility, and the pistil is more sensitive to heat stress than the pollen is [29]. This finding has also been confirmed in tomato, cotton, and corn [9]. During flowering, microspores are most sensitive to temperature changes during early development, and persistent high-temperature stress can lead to microspore abortion [30]. In cotton, average agronomic traits significantly decrease under high-temperature stress conditions, and the fiber becomes thicker [31]. Under high-temperature stress, the stomatal angle of leaves changes, and some stomata close, preventing increased transpiration rates from reducing the water content in the plant. This effectively reduces heat damage and can lead to smaller leaves [32]. To this end, we systematically investigated 13 key indicators via a two-year field experiment. PV, Chl, and LA represent the ability of plants to maintain physiological functions under high temperatures, whereas DBs, CB3, and DR reflect reproductive impairment and organ abscission caused by high temperatures. Population phenotypic variation analysis revealed that traits closely related to heat tolerance under high-temperature stress (such as Chl, LA, PV, DBs, FB3, CB3, and DR) presented high levels of variation (CV > 23.25%). In particular, the coefficient of variation for DBs was as high as 46.85%, indicating that these traits have rich genetic diversity within the population.

Crop breeding has evolved through different stages, from domestication to selection [33]. Early breeding methods primarily utilized the superior traits of wild germplasm for domestication and selection [34]. With the emergence of Mendelian inheritance, hybrid breeding through artificial selection, exemplified by the “Green Revolution” based on dwarfing techniques, emerged [35]. The Green Revolution achieved remarkable results in increasing rice, wheat, and maize yields, resulting in breakthroughs. With advances in molecular biology, genomics, and bioinformatics, new breeding technologies are rapidly developing [36]. Traditional hybrid breeding can still produce excellent cotton varieties, but the breeding efficiency is relatively low. MAS has absolute advantages in molecular breeding of crops such as rice, wheat, and corn [12]. On the basis of previous GWAS results, this study developed a KASP molecular marker at locus 5486185 on chromosome D06. We successfully genotyped 308 accessions, identifying two haplotypes, A/A and C/C, demonstrating the marker’s stable polymorphism and strong genotyping capabilities. The C/C haplotype significantly outperformed the A/A haplotype in several key heat tolerance traits (PV, LA, Chl, FB3) but presented significantly lower heat damage indicators (DBs, CB3, DR). Importantly, while significantly enhancing heat tolerance, the C/C genotype did not negatively impact key yield-component traits, such as EFB, NB, SBW, and LP, and even presented advantages in terms of pH and HFNFH. This finding suggests that this locus improves plant adaptability to high temperatures without incurring a yield penalty, resolving the common negative trade-off between stress tolerance and yield in stress-resistant breeding. This suggests that it may have potential applications in MAS, although further breeding practices are still needed. Notably, a few A/A haplotypes also exhibit heat resistance, such as Dai 4554, Xinluzhong 28, Xinluzhong 82, and some self-bred lines like 22L184 and NKY2018. This finding shows that different haplotypes at D06-5486185 can identify highly heat-resistant cotton. However, heat resistance is a quantitative trait controlled by multiple major and minor QTLs. Therefore, more markers are needed to comprehensively support breeding and production [5].

Another goal of molecular marker development is to identify closely linked major genes to facilitate production practices [37]. To this end, analysis revealed that the D06-5486185 locus is located in the intergenic region of two adjacent genes, *GH_D06G0408* and *GH_D06G0409*. These genes encode bZIP transcription factors, a family widely involved in plant responses to heat stress. Maize *bZIP60* activates the expression of the key heat shock protein *HSFTF13*, which in turn upregulates the expression of HSP genes, promoting tolerance to heat stress [38]. The winter melon *bZIP58* improves heat tolerance in transgenic *A. thaliana* lines [39]. In wheat, the bZIP members *TaOBF1-5B* interact with *TaHSP90* and *TaSTI*, increasing the expression of stress response genes and thus improving heat tolerance [40]. Overexpression of the cotton bZIP transcription factor *GhABF2D* enhances drought resistance through stomatal regulation [41]. Similarly, overexpression of *GhbZIP177* (*GhABF3*) improves the survival rate of cotton seedlings following drought stress treatments [42]. Expression analysis revealed that *GH_D06G0408* and *GH_D06G0409* were expressed at significantly higher levels in heat-tolerant accessions (C/C genotype) than in sensitive accessions (A/A genotype). The expression levels of these genes were significantly positively correlated with multiple heat-tolerant phenotypes (PV, Chl, FB3) and negatively correlated with heat-damaged phenotypes (DBs, CB3, DR). In summary, this study not only developed a KASP molecular marker that is tightly linked to heat tolerance in *G. hirsutum* and has no negative effects on yield traits but also provides a highly effective tool for identifying heat-tolerant genotypes and for MAS breeding. KASP markers are employed to genotype germplasm resources within breeding populations, including core collections, landraces, and exotic accessions. This approach enables the early identification of individuals carrying the target marker, thereby significantly reducing demands on field space, time, and labor costs while accelerating the overall breeding process [43]. Two key bZIP transcription factor genes were also identified, laying a solid foundation for elucidating the molecular mechanisms of heat tolerance in cotton. Future work will focus on validating the functions of these two genes via genetic transformation and gene editing techniques and further elucidating the molecular mechanisms regulating heat tolerance in *G. hirsutum*. The outcomes of functional validation enhance the reliability of KASP marker applications. Conversely, the utilization of KASP markers in breeding programs supplies novel candidate genes and informs new research directions for functional studies, thereby establishing a complete closed loop from laboratory research to field application and back again. This iterative process ultimately fosters the advancement of heat-tolerant cotton breeding.

## 5. Conclusions

In summary, this study characterized the heat tolerance phenotypes of 308 natural *G. hirsutum* populations over two consecutive years in the field. Using the KASP genotyping technique, we found that the D06-5486185 locus can distinguish different haplotypes. Combined haplotype and phenotype analysis revealed that the C/C haplotype presented excellent heat tolerance without negatively impacting key yield traits. Two bZIP transcription factor genes (*GH_D06G0408* and *GH_D06G0409*) were identified as candidate genes for heat tolerance in *G. hirsutum*. This molecular marker facilitates not only the early selection of breeding materials but also enables both foreground and background selection in backcross breeding programs. The candidate genes identified through this process can be directly utilized for functional validation and elucidating the mechanisms of heat tolerance, thereby significantly streamlining the conventional screening process. Thus, our study provides valuable molecular markers for assessing heat tolerance in *G. hirsutum* and serves as a resource for functional gene analysis and molecular breeding efforts.

## Figures and Tables

**Table 1:** Descriptive statistical analysis of 308 natural populations of *G. hirsutum*.

Trait	Mean	SD	Min	Max	Skew	Kurtosis	CV
PH	84.35	9.87	48.75	113.07	0.28	0.74	11.70%
HFNFH	30.70	3.24	19.60	41.45	0.27	0.67	10.55%
EFB	7.36	0.49	5.30	8.70	−0.74	1.73	6.66%
NB	6.54	0.85	2.40	11.25	0.93	5.48	13.00%
SBW	5.94	0.58	3.54	7.63	−0.22	1.54	9.76%
LP	42.21	2.78	31.28	48.52	−0.90	0.90	6.59%
PV	0.61	0.20	0.17	0.98	0.06	−0.73	32.79%
LA	84.42	19.68	47.29	152.25	0.85	0.33	23.31%
Chl	62.93	14.63	43.67	141.33	2.40	7.57	23.25%
DBs	2.54	1.19	0.20	8.40	0.91	1.54	46.85%
FB3	2.93	1.06	0.67	5.67	0.10	−0.66	36.18%
CB3	3.29	1.42	0.67	7.33	0.32	−0.67	43.16%
DR	43.42	12.70	8.33	87.50	0.13	0.10	29.25%

Note: Mean: average value, SD: standard deviation, Min: minimum, Max: maximum, Skew: skewness, CV: Coefficient of Variation.

## Data Availability

The original contributions presented in this study are included in the article/Supplementary Material. Further inquiries can be directed to the corresponding authors.

## Supplementary Materials

The following supporting information can be downloaded at https://www.mdpi.com/article/10.3390/genes16101154/s1. Table S1: All primers used in this study.
